# Polymerization of Allyltrimethylisilane and 4-Methyl-1-Pentene by Using Metallocene Catalysts

**DOI:** 10.3390/polym15092038

**Published:** 2023-04-25

**Authors:** Wei Wang, Minqiao Ren, Liping Hou, Shuzhang Qu, Xinwei Li, Zifang Guo

**Affiliations:** SINOPEC (Beijing) Research Institute of Chemical Industry Co., Ltd., No. 14 Beisanhuan Donglu, Chao Yang District, Beijing 100013, China

**Keywords:** metallocene, copolymerization, allyltrimethylisilane, 4-methyl-1-pentene, crystallization

## Abstract

Polymers of higher olefin, obtained by Ziegler-type polymerization, have been used in some critical fields, e.g., as the membrane for extracorporeal membrane oxygenation (ECMO), which plays an important role in the treatment of patients with severe COVID-19. The polymer obtained by a single-site catalyst, e.g., metallocene catalysts, demonstrated a higher performance. The homo- and co-polymerization of allyltrimethylisilane (ATMS) and 4-methyl-1-pentene (4M1P) were conducted using syndiospecific (cat 1) and isospecific (cat 2) metallocene catalysts. Cat 1 showed low conversions and provided a polymer with a higher molecular weight, while cat 2 behaved oppositely. ^13^C-NMR spectra certified the stereotacticity of the resultant polymer, and the resonance of the carbon atom of CH_2_ (αα’) between the two tertiary carbon atoms of the ATMS and 4M1P units were observed. This could be the evidence of the formation of a true copolymer. The crystallization of the polymer was explored using a differential scanning calorimeter (DSC) and wide angle X-ray diffraction (WAXD). All homopolymers and some of the copolymers showed high melting temperatures and low melting enthalpies. The WAXD patterns of the syndiotactic polymer and isotactic homopolymer or the ATMS-rich copolymer were consistent with the reported literature, but the isotactic 4M1P-rich copolymer provided the crystal form I, which is unusual for a 4M1P polymer without any pretreatment.

## 1. Introduction

Polyolefins are the most productive synthetic material in the world, including mainly polymers or copolymers of ethylene, propylene, and a small amount of 1-butene [1,2,3]. Among the coordination polymerization products made from olefins higher than butene, only poly(4-methyl-1-pentene) (PMP) has achieved industrialization [4]. This excludes synthetic oil, which is made from long chain α-olefins [5]. PMP demonstrates excellent optical transparency, electrical insulation properties, a high melting point, low surface tension, and a high permeability towards gases and general inertness and can therefore be used as an optical material [6], gas separation membrane [7,8,9], microelectronics and microsystems material [10], hydrogen storage material [11], blood contact material for medical apparatus [12], and so on. Isotactic PMP has a melting point of ~240 °C and a glass transition temperature of ~35 °C. However, there is usually a part of the polymer that does not crystallize. For common polymers, the mixture of crystalline and non-crystalline regions will result in anisotropy due to different densities. However, PMP has the lowest density of existing polyolefin materials, and the density of the crystalline and non-crystalline regions is very close (~0.830 g/cm^3^). As a result, light refracts very little when passing through the interface between the crystalline and non-crystalline regions of the PMP, resulting in excellent transparency. A hollow fiber membrane made of PMP can be used as gas separation membrane because of its good gas permeability and selectivity. For example, in ECMO, which is used to treat severe COVID-19 patients, the hollow fibers of the oxygenator are made of PMP [12]. As a result, the (co)polymerization of 4-methyl-1-pentene (4M1P) has been extensively studied [13,14,15,16]. The isospecific polymerization of 4M1P can be carried out through the coordination polymerization of Cr-, V-, Ti-, and Zr-based catalysts, of which the titanium catalyst is the most commonly used [13]. Among these, the supported, titanium-based Ziegler–Natta catalyst is the most commonly used catalyst and is also used in industrial production. It can produce the homopolymer of 4M1P and the copolymer of 4M1P and other α-olefins, and both are isotactic. However, the products of these catalysts are limited to this. It is powerless against the polymers with stereotacticity aside from isotacticity or copolymers containing other comonomers aside from α-olefins. From this point of view, single-site catalysts are more attractive [14,15,16,17]. They can not only produce polymers with more types of stereotactic structure, such as syndiotactic PMP [14,15,17], but can also incorporate more composed and structured comonomers into the polymer, such as a monomer containing an oxygen atom [16]. Using a metallocene catalyst combined with a methylaluminoxane (MAO) cocatalyst, we previously studied the isospecific copolymerization of 4M1P with α,ω-alkenols [16] and found that the long-chain comonomer (9-decen-1-ol) can significantly decrease the melting point of the copolymer, while the short-chain comonomer (4-penten-1-ol) has no such great effect. A possible reason for this is that the interference of the long-chain monomer on 4M1P unit crystallization is much stronger than that of the short-chain monomer. It was also found that the melting point of the copolymer increased slightly with the insertion of a small amount of short-chain α,ω-alkenols. We think that the hydrogen bond by the hydroxyl group could possibly help in increasing the melting point. On the other hand, the similar chain length of the short-chain α,ω-alkenol enables it to be included in the crystallizable segment of 4M1P units. However, there are a limited number of studies on higher olefins except for 4M1P [17,18,19]. For example, we used a syndiospecific metallocene to copolymerize allylcyclopentane with a variety of α-olefins [17]. Interestingly, we found that the melting point of the copolymer is closely related to the structure of the comonomer. All comonomers depressed the melting point of the copolymer. However, if the “shape” of the comonomer is similar to allylcyclopentane, such as allylcyclohexane or 4M1P, the effect is small. If the “shape” of the comonomer is very different from that of the allylcyclopentane, such as 1-decene, a copolymer with a much lower melting point will be obtained. The possible reason for this is that monomer units with similar structures can form a mixed crystal. This phenomenon can be called the “monomer similarity, crystal compatibility” of crystallization.

Allyltrimethylisilane (ATMS) is a type of monomer that can be used for coordination polymerization and is sometimes used as comonomer for ethylene or propylene copolymerization [20,21]. There are few studies on the homopolymerization of ATMS [22,23,24,25], which can obtain polymers with high melting point, and the study of the copolymerization of ATMS with other higher olefins is rare [25,26].

**Scheme 1:**
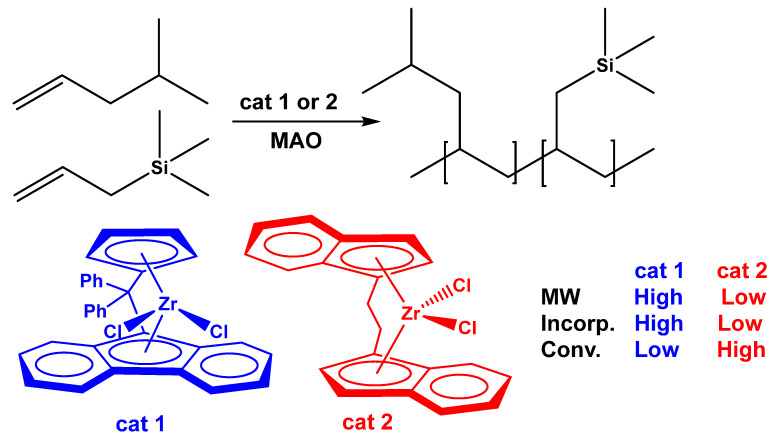
Copolymerization of ATMS with 4M1P.

As the best raw materials to date, the hollow fibers of PMP for ECMO oxygenators need to be treated with heparin to achieve an anticoagulant effect. However, PMP resin is strongly hydrophobic because it is only composed of carbon and hydrogen. This may lead to a low loading amount of heparin, and the heparin will be quickly lost in use. Therefore, modifying the coating of the PMP hollow fiber can improve its wettability and compatibility, which is helpful in improving the loading and firmness of heparin. Such a coating often uses silicon-containing substances [12]. Therefore, we hope to obtain the raw PMP resin for the coating-free or less coating fiber through the copolymerization of 4M1P and a silicon-containing monomer. The resin may be directly used in the hollow fiber of the oxygenator. Thus, we will have an interest in exploring the copolymerization of 4M1P with ATMS. At the same time, the structure and some basic properties of the copolymer, such as its crystallization behavior, are characterized and discussed. Due to the precise stereocontrollability of the polymerization of higher olefins and the excellent copolymerization ability of the metallocene catalyst [27,28,29], we herein report the homo- and copolymerization of ATMS and 4M1P with the use of isospecific (*rac*-EBIZrCl_2_) and syndiospecific (Ph_2_C(Cp)(Flu)ZrCl_2_) metallocene catalysts (see Scheme 1). The polymerization behavior was strongly affected by the catalysts used. The chemical composition and crystallization are further discussed.

## 2. Materials and Methods

All experiments were carried out under a nitrogen atmosphere in a Vacuum Atmosphere drybox or by using standard Schlenk techniques, unless otherwise specified. All chemicals used were of reagent grade and were purified through standard purification procedures. Toluene was distilled in the presence of sodium and benzophenone under a nitrogen atmosphere and was distilled and stored in a Schlenk tube in the drybox over molecular sieves. The metallocene catalysts, *rac*-ethylenebis(1-indenyl)zirconium dichloride (*rac*-EBIZrCl_2_) and diphenylmethylidene (cyclopentadienyl)(9-fluorenyl)zirconium dichloride (Ph_2_C(Cp)(Flu)ZrCl_2_), were purchased from Strem. The MAO solution in toluene (10 wt%) was purchased from Grace, and ATMS and 4M1P were obtained from TCI and were used as received. Other chemicals were also used as received.

Polymerization was conducted in toluene in a 250 mL glass reactor with an oil bath. The glass reactor was vacuumized and charged with a nitrogen atmosphere. Toluene and monomer(s) were introduced. The reaction mixture was heated to 50 °C, and the MAO solution and the catalyst solution in toluene were then injected to begin the polymerization. The mixture was stirred magnetically for a predetermined time. Subsequently, the mixture was poured into ethanol (~300 mL) containing concentrated hydrochloric acid (~10 mL). The resultant polymer was gathered on a filter paper by filtration and washed thoroughly with ethanol. It was then dried in vacuo at 60 °C for 24 h.

The molecular weight and molecular weight distribution (polydispersity index (pdi)) were determined using gel permeation chromatography (GPC) on a Waters Alliance GPCV2000 at 150 °C with 1, 2, 4-trichlorobenzene as the eluent.

The melting point of the polymer was determined on TAQ 100. Approximately 2 mg of the polymer sample was heated from room temperature to 300 °C with a heating rate of 10 °C per minute under a nitrogen atmosphere. After maintaining the temperature for 1 min, the sample was cooled down to room temperature, and the temperature was maintained for 1 min again. Then the sample was heated to 300 °C at a heating rate of 10 °C per minute, and the data were recorded.

Solution ^13^C NMR experiments were performed on a Bruker AVANCEIII-400 MHz spectrometer with a 10 mm PASEX ^13^C-^1^H/D Z-GRD probe. Sample solutions were prepared with approximately 200 mg of the polymer material dissolved in 2.5 mL of *d*_4_-*o*-dichrolobenzene (ODCB-*d*_4_) in a 10 mm NMR tube at 130 °C. All ^13^C NMR experiments were carried out at 125 °C, with a 20 Hz spinning rate, a 90° pulse angle, continuous Waltz-16 decoupling, a 120 ppm spectral width, a 5 s acquisition time, and a 10 s relaxation delay.

A wide-angle X-ray diffraction (WAXD) experiment was carried out on a Bruker D8 DISCOVER 2D X-ray diffractometer. The X-ray was generated using IμS micro Focus X-ray source incorporating a 50 W sealed-tube X-ray generator with a Cu target. The wavelength was 0.1542 nm. The power of the generator used for measurement was 45 kV and 0.9 mA. The X-ray intensities were recorded on a VÅNTEC-500 2D detector system with a pixel size of 136 μm × 136 μm. The distance from the sample to the detector was 198 mm. The spot size of the beam was 0.5 mm. The exposure time was 2 min. Polymer powder was used as generated without other treatment.

## 3. Results and Discussion

A syndiospecific metallocene catalyst, diphenylmethylidene (cyclopentadienyl) (9-fluorenyl)zirconium dichloride (Ph_2_C(Cp)(Flu)ZrCl_2_) (cat 1), and an isospecific metallocene catalyst, *rac*-ethylenebis(1-indenyl)zirconium dichloride (*rac*-EBIZrCl_2_) (cat 2), were used for the homo- and copolymerization of ATMS and 4M1P, and the results are summarized in Table 1. T_m_ and ΔH_m_ are the melting temperature and melting enthalpy of the polymers, respectively. M_w_ and PDI are the weight-average molecular weight and polymer dispersity index, respectively.

**Figure 1:**
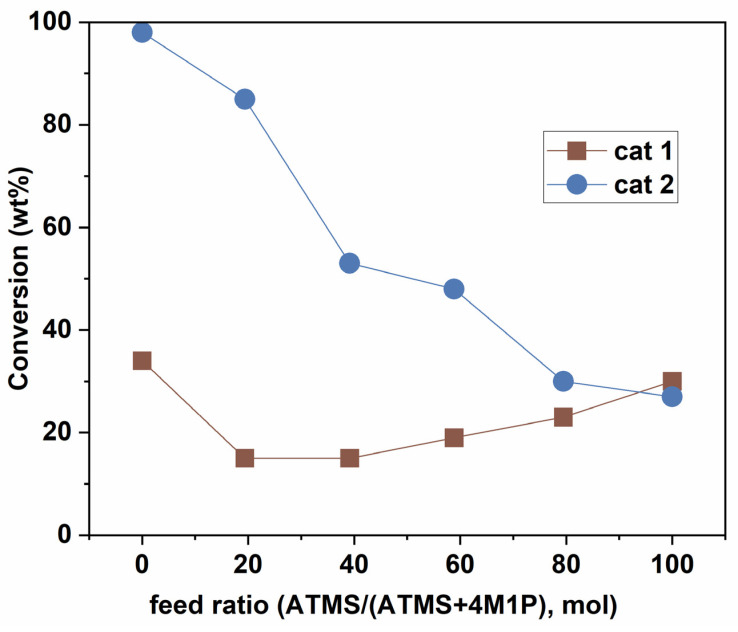
Influence of monomer feedings on weight conversion.

For both cats 1 and 2, the homopolymerization of 4M1P showed a higher conversion than that of ATMS. For the homopolymerization of 4M1P by cat 2 in particular, the monomer almost reached a quantitative conversion, within a polymerization time of 1 h. The introduction of ATMS into the monomer composition strongly impacted the overall conversion. From the data in Table 1 and the trend in Figure 1, it can be seen that the conversion of 4M1P reached 98% using cat 2, while ATMS only reached 27%. The depressing effect of ATMS on polymerization was also reflected in the copolymerization. With the increase in the proportion of ATMS in the monomer feeding, the conversion decreased monotonously. The conversion of polymerization using cat 1 showed an unusual trend. Although conversions of both monomers were not high (run 1: 34 wt%; run 6: 30 wt%), they were still higher than that of all copolymerization. All conversion formed a U-shaped trend as the monomer proportion changed.

All polymerizations provided the polymer with a narrow pdi (<2.0), indicating the single-site nature of the catalysts. The polymers produced by cat 1 had a higher molecular weight than the polymers produced by cat 2. For ethylene copolymerization, cat 2 always demonstrated a higher activity than cat 1 but provided the polymer with a lower molecular weight [30]. Here, the polymerization of 4M1P and ATMS showed the same behaviors as the ethylene polymerization, implying the intrinsic behaviors of these two catalysts, regardless of which monomer was involved. The catalytic activity and the molecular weight of the resultant polymer are closely related to the structure of the catalysts. Therefore, the observed differences are due to the difference in the structure of the catalysts. As shown in Table 2, the two catalysts are different in some structural parameters [31,32].

From the bond length data, we know that the zirconium atom of cat 2 is equidistant from the two indenyl ligands, while the zirconium atom of cat 1 is farther from the cyclopentadienyl ligand and closer to the flourenyl ligand. This may indicate that the flourenyl ligand provided the active site of cat 1 with greater steric hindrance, and thus suppressed not only the overall activity but also the chain transfer reaction. As a result, cat 1 provided a low conversion and resulted in a polymer with a high molecular weight. On the other hand, the metal atom of cat 2 is equidistant from the two ligands and has a medium steric hindrance between the cyclopentadienyl and flourenyl ligand of cat 1. This steric hindrance may cause greater resistance to the approach of a larger molecule (e.g., ATMS) to the active sites but less resistance to small molecules (e.g., 4M1P) or chain transfer agents (such as trimethylaluminum in the MAO solution). Thus, the use of cat 2 resulted in a higher activity, especially in the polymerization of 4M1P and the polymer with a lower molecular weight. Although the cyclopentadienyl ligand side of cat 1 is also conducive to the proximity of trimethylaluminum, the overall probability is less than that of cat 2 due to the steric hindrance from the flourenyl ligand. In general, from the perspective of bond length, due to steric hindrance, cat 2 has a higher activity but a lower activity for the polymerization of a larger monomer and the stronger chain transfer reaction, which resulted in the lower molecular weight of the polymer, while the opposite is true for cat 1.

According to the bond angle data, the large bond angle of ligand1-Zr-Ligand2 may make the ligand close to the position of the monomer coordination and chain propagation. This may hinder the larger molecule (e.g., ATMS) from approaching the active site but has little effect on the smaller molecules (e.g., 4M1P or trimethylaluminum). Thus, a larger Ind-Zr-Ind bond angle could explain why the polymer produced by cat 2 demonstrates a low molecular weight and a low ATMS incorporation. In addition, the larger bond angle of the bridge will make the active site more exposed, which is more favorable for the approach of the larger monomers. In this sense, cat 2 is more beneficial for the approach of ATMS and is prone to forming a copolymer with a higher ATMS incorporation.

**Figure 2:**
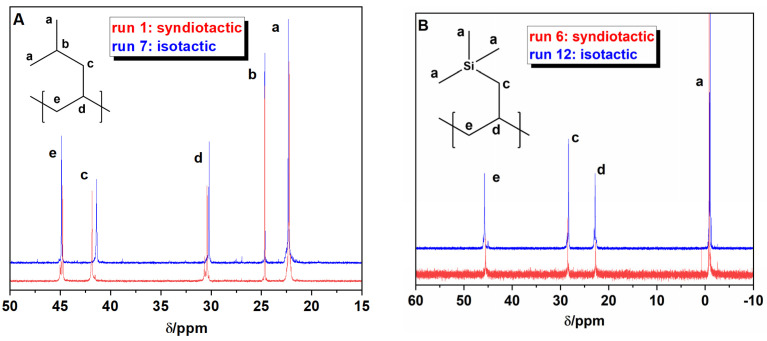
^13^C-NMR spectra of the isotactic or syndiotactic homopolymer of 4M1P (**A**) and ATMS (**B**).

Figure 2 showed the ^13^C-NMR spectra of poly(4M1P) and poly(ATMS) by cat 1 and 2, respectively [17,22,23,24,25,33]. The detailed attribution is summarized in Table 3.

**Figure 3:**
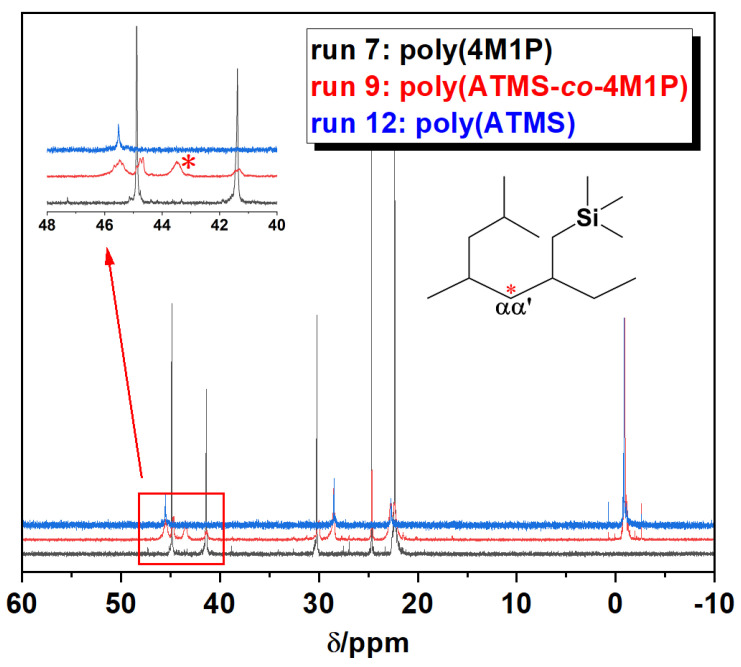
^13^C-NMR spectra comparison of homo- and copolymer.

Figure 3 compares the ^13^C-NMR spectra of the homo- and copolymers. All the resonance of the two homopolymers appeared in the spectra of the copolymer, and a new peak appeared at 43.49 ppm (asterisk). The new peak could be attributed to the αα’ carbon atom produced by the connection of two monomers, indicating the formation of a “true copolymer” rather than a blend of two homopolymers.

As the methyl carbon atoms connected with silicon atom show a very low chemical shift (−1.0 to −0.8 ppm) in the ^13^C-NMR spectrum, the incorporation of ATMS into the copolymer can be easily estimated. In addition to the resonances seen here, other resonances were provided by the carbon atoms of 4-methyl-1-pentene and three other carbon atoms of ATMS. The integral intensity of the NMR spectrum has the following relationship with ATMS incorporation (x).

$$\left\{\begin{array}{l} I_{Si}=3xa\\ I_{other}=3xa+6(1-x)a \end{array}\right.$$

where *I_Si_* is the integral intensity of the carbon atoms of three methyl groups connected with silicon atom, and *I_other_* is the integral intensity of the other atoms.

After transformation, the incorporation of ATMS into the copolymer can be calculated by the following formula.

$$x=\frac{2I_{Si}}{I_{other}+I_{Si}}$$

According to this method, the incorporation of ATMS is calculated and listed in Table 1. Although the NMR determination of some samples was not carried out successfully due to the problem of solubility, the available data still show some trends. For the three copolymers (runs 4, 9, and 10) with NMR results, the incorporation of ATMS into the polymer was higher than the ratios in the feed (incorporation vs. ratio in feed: run 4, 89.0 vs. 58.8%; run 9, 66.0 vs. 39.2%; run 10, 81.5 vs. 58.8%). This means that under the mixed monomer conditions of ATMS and 4M1P, it is easier for the former to participate in polymerization than the latter. The homopolymerization activity of ATMS was significantly lower than that of 4M1P. Additionally, the introduction of ATMS significantly decreased the activity during copolymerization. Considering these two observations, it can be inferred that ATMS has a higher coordination probability but a slower insertion rate than 4M1P. For the two polymerizations with the same feed ratio but different catalysts, the ATMS incorporation of the obtained polymers were different (runs 4 vs. 10). The copolymer (run 4) with a higher ATMS incorporation was obtained from the syndiospecific catalyst. For any copolymerization, if the bulkier monomer is regarded as the comonomer, it is reasonable to believe that cat 1 is a better catalyst than cat 2 if only the copolymerization is considered [30].

The melting point of all homopolymers was detected by DSC. The melting point of the isotactic polymer was higher than that of syndiotactic polymer, and the melting point of the ATMS polymer was higher than that of the 4M1P polymer. This is generally consistent with the literature [22,33]. However, the melting point obtained in the earlier research was higher than the melting point in this study, which may be related to the specific catalysts. Although the polymer showed a high melting point, its melting enthalpy was not high, and it was also lower than the reported value in the previous literature [22].

**Figure 4:**
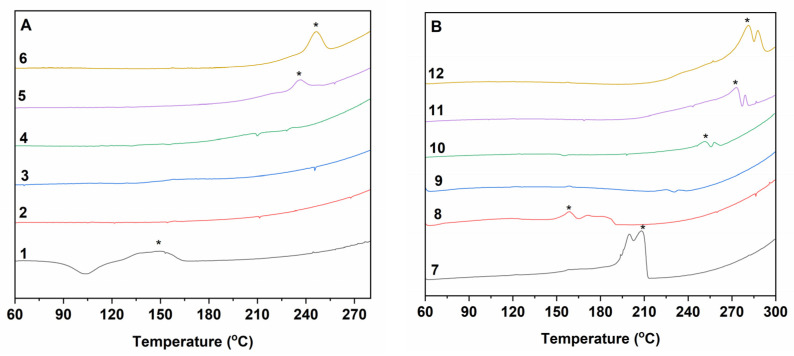
DSC melting curves for syndiotactic (**A**) and isotactic (**B**) polymers (main peak is marked with an asterisk).

As can be seen in Figure 4A, the syndiotactic PMP showed a melting temperature of 149 °C, while a melting temperature of 246 °C for the syndiotactic P(ATMS) was observed. It is notable that there was an exothermic peak around 104 °C during the second heating process of PMP (sample 1). This could be attributed to the cold crystallization in the heating process, resulting from an incomplete crystallization while cooling, which was observed for either the polyolefin [34] or non-olefin polymer [35]. No melting point could be detected for samples 2, 3 and 4, even though the sample 4 incorporated an 89 mol% ATMS unit. The isotactic polymers exhibited a higher melting temperature, but their melting enthalpies were lower than their syndiotactic analogies (Figure 4B). This may be due to the great steric hindrance caused by the chain end of the branch, which makes the crystallization of the isotactic polymer loose, resulting in the low melting enthalpy. This may also be the reason why the isotactic PMP density is very low (~0.830 g/cm^3^). Until now, the density of PMP is the lowest among commercial polyolefin materials. The syndiotactic polymer tends to form a relatively dense crystal due to the staggered distribution of the branch. Thus, its melting enthalpy is obviously higher than that of the isotactic polymer.

**Figure 5:**
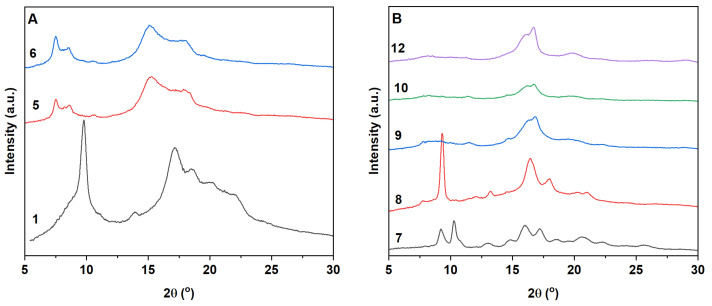
Wide-angle X-ray diffraction patterns of as-polymerized samples ((**A**) syndiotactic; (**B**) isotactic).

The Wide-angle X-ray diffraction (WAXD) patterns of some selected, as-polymerized samples are shown in Figure 5, in order to explore more structural detail of the polymer.

In Figure 5A, the WAXD patterns of the syndiotactic homopolymer of 4M1P (run 1) or ATMS (run 6) were in accordance with the Bragg distance of reflection reported in the literature [22,36], while the crystal structure of the copolymer (run 5) was similar to the syndiotactic ATMS homopolymer (run 6). This may be due to the exclusion of a small amount of the 4M1P unit from the crystal of the syndiotactic homopolymer of ATMS. Similar observations have been reported in crystallization research on other polyolefins [37,38,39].

In Figure 5B, the WAXD patterns of the isotactic ATMS homopolymer and copolymers (runs 9, 10, and 12) were similar and demonstrated the typical crystal structure of an isotactic ATMS homopolymer [22].The WAXD of run 7 exhibited form II of isotactic PMP. It is slightly surprising that the WAXD of run 8 showed form I of isotactic PMP [40,41]. To our best knowledge, form I of isotactic PMP can only be obtained from melts or some specific solutions [13]. We previously showed that the 4M1P polymer with a high incorporation of α,ω-alkenol was detected as crystal form I [16]. In the present work, we found that the incorporation of ATMS can play the same role; however, the mechanism remains unclear.

## 4. Conclusions

In this work, we have shown the homo- and copolymerization of 4M1P and ATMS by using two metallocene catalysts with different stereospecificities. Homopolymers of each monomer and their copolymer with syndiotacticity and isotacticity were obtained. The ^13^C-NMR test proved the difference between the polymers with different stereotacticities and demonstrated their efficient copolymerization. The activity depended on both the monomer type and the catalyst used. Both 4M1P and the isospecific catalyst, *rac*-EBIZrCl_2_, are prone to achieving the high activity, while ATMS and the syndiospecific catalyst, Ph_2_C(Cp)(Flu)ZrCl_2_, behave oppositely. On the other hand, the molecular weight of polymers obtained using syndiospecific catalysts, whether homopolymers or copolymers, is much higher than the molecular weight obtained by isospecific catalysts. However, cat 1, with the asymmetric steric hindrance, showed better selectivity to larger monomer ATMS, thus obtaining the copolymer with the higher incorporation of ATMS. These performances are closely related to the molecular structure of the catalysts. The chemical environment caused by the steric and electronic effects of the ligands may play a decisive role. This may inspire researchers to precisely design the ligand with suitable steric and electronic effects so as to optimize the specific catalytic behavior or maximize the overall performance, for example, of a specific catalyst for a specific product. In this way, polyolefin products meeting the end-use requirements can be obtained.

The DSC provided the results of a high melting temperature and low enthalpy for all polymers. The syndiotactic homopolymers demonstrated melting points of 149 and 246 °C, while the isotactic homopolymers demonstrated higher melting points of 208 and 281 °C for 4M1P and ATMS, respectively. The melting points of their copolymers were between those of the homopolymers. Additionally, WAXD exhibited predictable patterns for the homopolymers, while isotactic poly(4M1P-*co*-ATMS) provided an unusual crystallization form. We would be interested to explore this mechanism in the future in more detail.

## Figures and Tables

**Table 1:** Homo- and copolymerization and microstructural characterization of ATMS and 4M1P by using metallocene catalysts ^a^.

Run	ATMS (mmol)	4M1P (mmol)	Polymer (g)	Conv (wt%)	T_m_ ^b^ (°C)	ΔH_m_ ^b^ (J/g)	M_w_ ^c^ (k)	pdi ^c^	ATMS Incorp. ^d^ (mol%)
1	0	19.7	0.56	34	149	8.3	26.5	1.90	0
2	3.8	15.8	0.28	15	-- ^e^	-- ^e^	25.4	1.85	-- ^f^
3	7.6	11.8	0.30	15	-- ^e^	-- ^e^	37.3	1.78	-- ^f^
4	11.3	7.9	0.41	19	-- ^e^	-- ^e^	28.8	1.80	89.0
5	15.1	3.9	0.55	23	236	2.7	34.6	1.84	-- ^f^
6	18.9	0	0.77	30	246	6.8	46.3	1.83	100
7	0	19.7	1.62	98	208	45.1	8.5	1.94	0
8	3.8	15.8	1.56	85	159	25.2	4.7	1.69	-- ^f^
9	7.6	11.8	1.15	53	-- ^e^	-- ^e^	5.0	1.71	66.0
10	11.3	7.9	0.96	48	251	1.7	6.2	1.85	81.5
11	15.1	3.9	0.71	30	273	16.5	7.5	1.69	-- ^f^
12	18.9	0	0.69	27	281	19.4	6.1	1.75	100

^a^ catalyst 5.0 μmol, runs 1–6 cat 1, Ph_2_C(Cp)(Flu)ZrCl_2_; runs 7–12, cat 2, *rac*-EBIZrCl_2_; MAO 10.0 mmol (Al/Zr = 2000), 60 min, 50 °C, in toluene, total toluene volume 20 mL. ^b^ determined by DSC; some are bimodal. ^c^ determined by GPC. ^d^ determined by ^13^C-NMR. ^e^ no melting point could be detected. ^f^ NMR experiment could not be carried out due to the bad solving condition.

**Table 2:** Some structural parameters of cats 1 and 2.

	Ph_2_(Cp)(Flu)ZrCl_2_ [31] (cat 1)		*rac*-EBIZrCl_2_ [32] (cat 2)	
Bond length (Å)	Zr-Cl	2.424(2)	Zr-Cl	2.3884(5)
	Zr-C(1)	2.417(8)	Zr-C(1)	2.438(2)
	Zr-C(2)	2.513(6)	Zr-C(2)	2.443(2)
	Zr-C(3)	2.680(6)	Zr-C(3)	2.531(2)
	Zr-C(27)	2.452(8)	Zr-(C3a)	2.624(2)
	Zr-C(28)	2.450(6)	Zr-(C7a)	2.553(2)
	Zr-C(29)	2.523(6)		
Bond angles (°)	Flu-Zr-Cp	118.2	Ind-Zr-Ind	125.3(1)
	Cl-Zr-Cl	95.6	Cl-Zr-Cl′	99.09(3)
	C1-C14-C27	99.6	C(1)-C(8)-C(8′)-C(1′)	45.6(3)
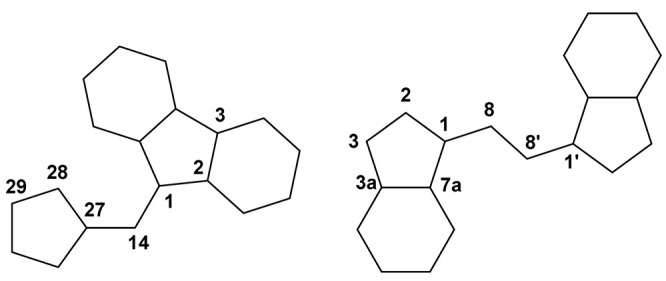				

**Table 3:** Chemical shifts of isotactic or syndiotactic homopolymers of 4M1P and ATMS.

Polymer	Chemical Shift of Carbon (ppm)				
	e	c	d	b	a
iso-PMP	44.84	41.38	30.19	24.68	22.33
syndio-PMP	44.75	41.80	30.36	24.67	22.25
iso-PATMS	45.47	28.48	22,72	--	-0.89
syndio-PATMS	45.70	28.25	22,78	--	-1.07

## Data Availability

Not applicable.
